# A Case of a Large Intraabdominal Abscess in a Patient with Cirrhosis Misdiagnosed as Spontaneous Bacterial Peritonitis

**DOI:** 10.1155/2022/5951115

**Published:** 2022-10-05

**Authors:** Saeed Ali, Abdullah Sohail, Kyle Brown

**Affiliations:** ^1^Division of Gastroenterology & Hepatology, Department of Medicine, University of Illinois at Chicago, Chicago, USA; ^2^Department of Internal Medicine, University of Iowa Roy J. and Lucille A. Carver College of Medicine, Iowa City, IA, USA

## Abstract

Spontaneous bacterial peritonitis is a known complication of patients with decompensated cirrhosis and ascites. It is differentiated from secondary bacterial peritonitis by the absence of an intraabdominal source of infection. We present a 56-year-old man with alcoholic cirrhosis who underwent multiple paracenteses that yielded fluid with progressively increasing neutrophil counts and several different organisms, recurring despite numerous treatments for SBP. Eventually, a computed tomography (CT) of the abdomen and the pelvis revealed a large intraabdominal abscess (22 × 13 cm) treated with an ultrasound-guided drain and IV antibiotics. Recurrent episodes of SBP despite appropriate antibiotics should raise suspicion for secondary bacterial peritonitis. It is crucial to differentiate SBP from secondary bacterial peritonitis as the mortality of the latter is much higher without prompt treatment. Appropriate antibiotic regimens, prompt surgical treatment, and postoperative care are crucial to improving clinical outcomes in these patients.

## 1. Introduction

Spontaneous bacterial peritonitis (SBP) is diagnosed by positive ascitic fluid bacterial culture or an absolute polymorphonuclear leukocyte (PMN) count ≥250 cells/mm^3^ or both. SBP is an ascitic fluid infection without an intraabdominal surgically treatable source [1]. It results from changes in the intestinal microbiota, altered intestinal permeability, bacterial translocation, and systemic immune dysfunction [2]. It is essential to differentiate SBP from secondary bacterial peritonitis as the mortality of the latter is exceptionally high if treated alone with antibiotics without appropriate surgical intervention [3]. SBP is almost always monomicrobial, and the growth of more than one organism should raise suspicion for secondary bacterial peritonitis. We present a case of a patient with ascites who presented with recurrent polymicrobial peritonitis despite antibiotic treatment and was eventually found to have a large intraabdominal abscess.

## 2. Case Presentation

A 56-year-old man with alcoholic cirrhosis and diabetes mellitus presented approximately six months earlier with abdominal distention at an outside hospital. An abdominal computed tomography (CT) showed ascites and a cirrhotic-appearing liver with no other abnormalities. The ascitic fluid contained 36 polymorphonuclear leukocytes (PMNs)/mm^3^, and cultures showed no growth. Shortly after that, he underwent a screening colonoscopy, during which a 4 cm cecal tubulovillous adenoma was removed. His ascites initially responded to diuretics and reduced alcohol consumption, but abdominal distention recurred four months after the first tap. A repeat paracentesis removed 6 liters (*L*) of cloudy amber fluid with 2648 PMNs/mm^3^; cultures grew no organisms, and no antibiotics were prescribed. Eleven days later, the ultrasound showed complex loculated ascites. Subsequent paracentesis yielded 1.5 L of yellowish-milky fluid with 28,292 PMNs/mm^3^, and the cultures grew *Klebsiella oxytoca* and *Streptococcus salivarius*. The patient was diagnosed with SBP, admitted, and treated with IV cefotaxime for four days. A paracentesis three days after admission removed 1.4 L of tan-cloudy fluid, with >3 billion nucleated cells/mm^3^, 90% PMNs, and the cultures grew the same two organisms. A comparison of the ascites fluid analysis is presented in Table 1. He improved symptomatically and was discharged on cefixime for five days, after which he was instructed to begin trimethoprim/sulfamethoxazole for SBP prophylaxis. Two weeks later, while on SBP prophylaxis, paracentesis removed 4 L of cloudy-green fluid, containing nearly a million nucleated cells/mm^3^; cultures grew *Streptococcus anginosus*.

**Figure 1:**
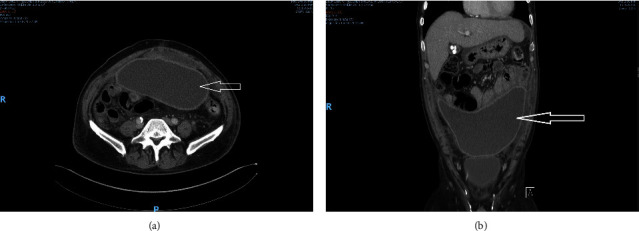
(a) Computed tomography (CT) abdomen/pelvis cross sectional view showing rim-enhancing fluid collection in the anterior peritoneal cavity measuring 22 × 13 × 7.2 cm representing intraabdominal abscess. (b) Computed tomography (CT) abdomen/pelvis coronal view showing rim-enhancing fluid collection in the anterior peritoneal cavity measuring 22 × 13 × 7.2 cm representing intraabdominal abscess.

**Figure 2:**
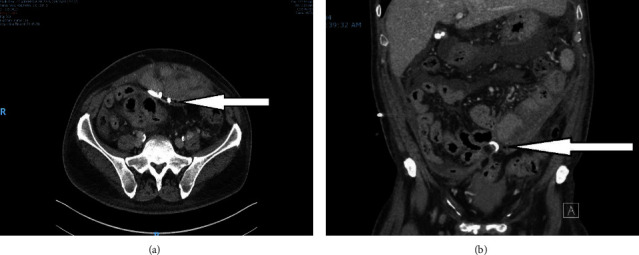
(a) Computed tomography (CT) abdomen/pelvis cross sectional view showing near complete resolution of the previously large intraabdominal abscess with small amount of air visible along the track of the drain catheter (solid white arrow). (b) Computed tomography (CT) abdomen/pelvis coronal view showing near complete resolution of the previously large intraabdominal abscess with air visible along the track of the drain catheter (solid white arrow).

Four weeks later, he presented to our institution from an outside hospital, complaining of abdominal pain, distention, weight loss, and poor appetite. He was afebrile with stable vital signs; the exam was remarkable for mass-like firmness of the abdomen. WBC count was normal (9.6 × 10^3^/mm^3^) with normal neutrophil count and mildly elevated bands (582/mm^3^), hemoglobin was 8.6 g/dL, bilirubin and liver enzymes were normal. A CT abdomen/pelvis with contrast revealed a large (22 × 13 cm) intraabdominal abscess (Figures 1(a) and 1(b), from which 5.6 L of milky-white fluid was drained, which grew *Streptococcus anginosus* and *Lactobacillus fermentum*. An ultrasound-guided drain was placed, and the patient was discharged on home IV antibiotics. He returned three weeks later, feeling much better. A repeat CT scan revealed near-complete resolution of the abscess (Figures 2(a) and 2(b). The drain and IV line were removed, and he was switched to oral amoxicillin for an additional three weeks. The patient was discharged and did well afterward.

## 3. Discussion

Ascites is a major complication of cirrhosis [4] and a poor prognostic indicator with 50% mortality in 2 years [5]. Diuretics and salt restriction are the mainstays of treatment, but large-volume paracentesis (LVP) is safe and effective in patients with treatment-resistant ascites [6]. Randomized controlled trials showed that patients treated with repeated LVP followed by volume expansion have shorter length of stay, preserved systemic hemodynamics and renal function, decreased risk of spontaneous bacterial peritonitis (SBP), and less frequent development of hepatic encephalopathy than patients receiving diuretics alone [7]. The complications of paracentesis are rare but include bowel perforation, infection, bleeding, and ascitic fluid leakage from the puncture site [8].

Bacterial peritonitis is a common complication in patients with ascites. Its prevalence was reported at around 12% in 2001 but is steadily declining with the widespread use of antibiotics for prophylaxis [9]. Gram-negative bacteria are the culprits in up to 80% of culture-positive cases of SBP [10], where *E. coli*, *Streptococci*, and *Klebsiella* are the most frequent isolates [11].

This is an unusual case of a large intraabdominal abscess misdiagnosed as SBP. When therapy for SBP fails and ascitic fluid PMN counts remain elevated, this should raise the suspicion of secondary bacterial peritonitis. Secondary bacterial peritonitis due to a surgically treatable source like perforation or inflammation of an intraabdominal organ can mimic SBP [11].

Ascitic fluid characteristics of secondary bacterial peritonitis were present in our patient, including a very high PMN count and polymicrobial cultures. Other criteria that can help distinguish spontaneous from secondary peritonitis include total protein >1 g/dL, lactate dehydrogenase > upper limit of normal for serum, and glucose <50 mg/dL [12], but these were not obtained in our patient. Follow-up PMN counts 48 hours after treatment can also help identify secondary peritonitis cases. The 48-hour PMN count is almost always below the pretreatment level in SBP treated with appropriate antibiotics [13], but rises despite treatment in nonperforation secondary peritonitis [14]. In our case, the progressive and dramatic increase in PMNs and the number of organisms cultured from the fluid were clues that this was not an uncomplicated case of SBP. These findings should have prompted cross sectional imaging to evaluate for an intraabdominal source of infection, but this was not done until several weeks into his course.

The source of the abscess is not clear in our patient. At the onset of this illness (his second tap, four months after the initial one), the paracentesis showed a high PMN count with negative cultures. The patient was not treated with antibiotics, and a week and a half later, the fluid was grossly purulent, with a 10-fold increase in PMNs and grew two organisms. Given that the PMN count was already elevated on the preceding paracentesis, it seems unlikely that the subsequent culture-positive infection was caused by bowel injury during the earlier tap. It is also doubtful that the abscess was itself a manifestation of SBP since the presence of multiple organisms is not typical of SBP; furthermore, we found no instances in the literature of abdominal abscess arising from SBP. The possibility that the abscess resulted from either a microperforation caused by the cecal polypectomy or bowel puncture during the initial paracentesis seems remote since those procedures were done months before the onset of symptoms; however, given his indolent course and lack of systemic toxicity even when presented with a large abscess, this cannot be excluded entirely.

This case illustrates the importance of recognizing that not all cases of neutrophilic ascites in cirrhosis represent SBP. The lack of recognition of ascitic fluid findings that differentiate SBP from secondary bacterial peritonitis led to a delay in diagnosing a huge intraabdominal abscess. Fortunately, the patient eventually received definitive treatment and recovered well.

## Figures and Tables

**Table 1:** Comparison of the ascite fluid analysis.

Date of paracentesis	5/11/2016^*∗*^^*∗∗*^	29/09/2016	10/10/2016	13/10/2016	12/10/2016^*∗∗*^
Total nucleated cell count/mm3	522	3,153	1,57,180	3,44,44,30,000	5,85,100
Polymorphonuclear leukocytes (PMNs)/mm3	36	2,648	28,292	3,09,99,87,000	
Peritoneal fluid color	Yellow	Amber	Yellow	Tan	Yellow
Peritoneal fluid culture	No growth	No growth	*Klebsiella oxytoca* and *Streptococcus salivarius*	*Klebsiella oxytoca* and *Streptococcus salivarius*	*Streptococcus anginosus* and *Lactobacillus fermentum*

^∗∗∗^Date of diagnosis of cirrhosis. ^∗∗^Date of diagnosis of abscess.
